# Metabolomics identifies chenodeoxycholic acid as a protective factor in diabetic foot ulcers

**DOI:** 10.3389/fendo.2026.1754743

**Published:** 2026-02-13

**Authors:** Ying Li, Ran Guo, Lu Lv, Tao Jiang, Chenning Zhang, Jiangying Chang, KeYan Hu, Huifang Peng, Lulu Chen, Ran Chen, Hetao Chen, Yujin Ma

**Affiliations:** 1 Department of Pharmacy, The First Affiliated Hospital, College of Clinical Medicine of Henan University of Science and Technology, Luoyang, China; 2 Luoyang Key Laboratory of Transplantation and Immunological Studies for Hematological Diseases, Department of Clinical Laboratory, The First Affiliated Hospital, and College of Clinical Medicine of Henan University of Science and Technology, Luoyang, China; 3 Henan Key Laboratory of Rare Diseases, Endocrinology and Metabolism Center, The First Affiliated Hospital, and College of Clinical Medicine of Henan University of Science and Technology, Luoyang, China; 4 Department of Pharmacy, Xiangyang No.1 People’s Hospital, Hubei University of Medicine, Xiangyang, China

**Keywords:** chenodeoxycholic acid, diabetic foot, fibroblasts, functional metabolomics, protective factors

## Abstract

**Objective:**

Diabetic foot ulcers (DFU) are a severe complication with high amputation and mortality rates, involving profound metabolic dysregulation. Current treatments lack interventions targeting the metabolic microenvironment. Chenodeoxycholic acid (CDCA) regulates glucose/lipid metabolism and inflammation, but its role in DFU remains unknown. This study aims to identify key metabolites in the serum of patients with diabetic foot ulcers. For the first time, it focuses on and identifies CDCA, a key bile acid, as an endogenous protective factor, and validates the biological role of CDCA in promoting wound healing, thereby providing a foundation for novel therapeutic strategies targeting the “metabolic microenvironment.”

**Methods:**

Untargeted metabolomics (UHPL-MS/MS) was performed on serum from 18 healthy controls, 18 diabetes mellitus (DM) patients, and 18 DFU patients. Multivariate statistics and logistic regression were used to identify differential metabolites and protective factors. *In vitro*, human skin fibroblasts under high glucose (30 mM) were treated with CDCA (5–25 nM). Proliferation, migration, and repair gene expression were assessed.

**Results:**

Clinical evaluation revealed that DF patients exhibited more pronounced systemic inflammation (elevated hs-CRP, ESR, and WBC), coagulation abnormalities (increased fibrinogen and D-dimer levels), and higher prevalence of vascular complications compared to other groups. Metabolomic analysis identified 41 significantly altered metabolites between the DM and DF groups, among which CDCA was markedly downregulated in the DF group (fold change = 0.66, VIP = 2.09, *P* = 0.008). Logistic regression analysis confirmed CDCA as an independent protective factor against DFU (OR = 0.429, 95% CI: 0.225–0.815, *P* = 0.010). *In vitro* studies demonstrated that CDCA dose-dependently reversed high glucose-induced impairment of HSF function, significantly enhancing cell proliferation and migration, and upregulating mRNA expression of PCNA, α-SMA, and Vimentin.

**Conclusion:**

This study identifies CDCA as a key protective metabolite in DF. Reduced serum CDCA levels are independently associated with increased risk of DF. Functional evidence confirms that CDCA mitigates high glucose-induced fibroblast dysfunction and promotes wound repair processes. Targeting the CDCA signaling pathway or supplementing CDCA may represent a novel therapeutic strategy for DF by remodeling the “metabolic microenvironment”.

## 1 Introduction

Diabetic foot (DF) is defined as wounds that manifest on the feet of individuals suffering from type 1 or type 2 diabetes (1). It is typified by infections, ulcers, and/or deep - tissue damage stemming from peripheral neuropathy and vascular complications in the lower limbs of diabetic patients. The pathogenesis of DF is intricate and multi-factorial, encompassing interrelated mechanisms like infection, micro-and macrovascular diseases, and neurological impairment (2). Globally, patients suffering from diabetic foot ulcers (DFU) encounter a five - year mortality rate that is as high as 34%. Moreover, non - traumatic lower limb amputations caused by this condition make up more than half of all cases, which poses a significant public health challenge (3, 4). Current clinical management guidelines, such as those recommended by the International Working Group on the Diabetic Foot, mainly focus on infection control, revascularization for ischemia, and mechanical offloading. Although these interventions are effective in saving limbs in critical situations, they frequently fail to fundamentally tackle the root challenge of chronic non-healing wounds (5, 6).

DFUs represent the most severe chronic complication of diabetes, with their pathophysiology primarily rooted in impaired wound healing caused by hyperglycemia-induced tissue damage (7, 8). A growing body of evidence indicates that persistent hyperglycemia creates an unfavorable microenvironment, which impairs re-epithelialization, angiogenesis, and extracellular matrix remodeling via multiple mechanisms, including the accumulation of advanced glycation end products, heightened mitochondrial oxidative stress, and continuous inflammatory responses (9, 10). Therefore, going beyond the traditional paradigms of “anti-infection” and “vascular patency”, exploring the metabolic basis of this pathological microenvironment, and identifying key endogenous regulatory factors that can restart the normal repair process have become the new frontier in surmounting current therapeutic limitations.

Metabolomics, as a scientific discipline, systematically characterizes the compositional profiles and dynamic changes of endogenous small-molecule metabolites within biological systems. The analytical targets of metabolomics span a broad spectrum of endogenous and environmentally-derived low-molecular-weight compounds, such as sugars, lipids, amino acids, fatty acids, alkaloids, and polyphenols (11). Metabolomics, through the utilization of high-throughput analysis to monitor the dynamic changes in endogenous metabolites after physiological or pathological stimuli, offers a direct portrayal of the organism’s actual physiological and biochemical state. This, in turn, facilitates the identification of potential diagnostic biomarkers and therapeutic targets (12, 13). In studies of exudate from DFU, metabolomics has identified multiple key small molecules, such as betaine, lactic acid, and carnitine, which play diverse roles in wound healing. These roles include enhancing cellular function, exerting anti-inflammatory and antioxidant effects, and promoting angiogenesis (14, 15). In the field of diabetes and its complications, existing research has indicated that abnormal changes in metabolic profiles, such as those of amino acids, fatty acids, and bile acids in blood or tissues, are closely related to insulin resistance, vascular endothelial dysfunction, and chronic inflammation (16–18). Bile acids, particularly primary bile acids like chenodeoxycholic acid (CDCA), are capable of activating key nuclear receptors and membrane receptors, including the farnesoid X receptor (FXR) and the G protein - coupled bile acid receptor (GPBAR1/TGR5). As a result, they can precisely regulate glucose homeostasis, energy expenditure, innate immune responses, and inflammatory pathways (19–21). There is compelling evidence suggesting that disruptions in bile acid metabolism are closely linked to the initiation and advancement of type 2 diabetes and non-alcoholic fatty liver disease (22, 23). Research has demonstrated that CDCA can enhance insulin sensitivity in both hepatic and peripheral tissues and exert anti-inflammatory effects (24). Although the association between perturbations in bile acid metabolism and the pathophysiology of systemic diabetes has been tentatively established, it remains uncertain whether bile acids play a crucial role in the pathological progression of DFU, which is characterized by severe local tissue damage. Specifically, it is unclear regarding the relationship between altered serum bile acid levels and ulcer risk, or whether bile acids can directly regulate wound repair processes. Numerous bioactive molecules, including chenodeoxycholic acid (CDCA), face significant challenges in complex wound environments due to limited stability, poor membrane permeability, and inadequate targeting capability. Advanced drug delivery systems—such as solid lipid nanoparticles (SLNs) that enhance dermal penetration, cubic micelles capable of co-delivering multiple therapeutic agents, and highly deformable transfersomes—present promising strategies to overcome these physiological and biochemical barriers (25, 26). By improving the local retention and enabling controlled release of active compounds, these delivery platforms hold potential to translate the pharmacological efficacy of agents like CDCA into measurable clinical outcomes, representing a key focus for future translational research within this study.

This study conducted an exploratory investigation by integrating clinical metabolomics with functional validation. Through the application of untargeted metabolomics technology, the serum metabolic profiles of patients with DFU were comprehensively characterized, and key metabolites associated with disease progression were precisely identified. Simultaneously, bioinformatics analyses and statistical modeling were utilized to accurately pinpoint endogenous metabolites with potential protective effects. The regulatory effects of candidate molecules on the functions of wound-repair cells were validated using high-glucose cellular models. The aim was to uncover novel mechanisms underlying DFU and provide a theoretical basis for the “metabolic microenvironment remodeling” therapeutic strategy.

This study was carried out as a case-control investigation in strict compliance with the principles of the Declaration of Helsinki and obtained ethical approval from the Ethics Committee of the First Affiliated Hospital of Henan University of Science and Technology (approval number: 2024 - 03 - K0048). The registration number at the Chinese Clinical Trial Registry (ChiCTR) is ChiCTR2400088055. Written informed consent was acquired from all participants before their enrollment, and the confidentiality of personal data was rigorously maintained throughout the study.

## 2 Materials and methods

### 2.1 Research subjects and clinical data collection

#### 2.1.1 Grouping of research subjects and inclusion/exclusion criteria

The case group comprised 18 patients diagnosed with diabetic foot (DF group) and 18 patients with diabetes mellitus but without foot complications (DM group), who were hospitalized in the Department of Endocrinology at the First Affiliated Hospital of Henan University of Science and Technology between January 2023 and December 2024. The control group consisted of 18 healthy individuals selected from the hospital’s routine physical examination population, matched for age and sex (HA group). The diagnosis and severity classification of DF were established based on the joint guidelines of the International Working Group on the Diabetic Foot (IWGDF) and the Infectious Diseases Society of America (IDSA), with six patients classified as DF grade 2, six as DF grade 3, and six as DF grade 4.

Inclusion criteria:

DF group: (1) Diagnosis consistent with the latest definition of DF outlined in the Clinical Pathway for the Diagnosis and Treatment of Diabetic Foot in China (2023 Edition); (2) Age ≥ 18 years; (3) Willingness to participate and provision of written informed consent.

DM group: (1) Fulfillment of diagnostic criteria for type 2 diabetes as defined in the Chinese Guidelines for the Prevention and Treatment of Type 2 Diabetes (2020 Edition); (2) Age ≥18 years; (3) Absence of DF or other severe diabetic complications (e.g., proliferative retinopathy, end-stage renal disease); (4) Willingness to participate and provision of written informed consent.

HA group: (1) No personal or family history of diabetes mellitus; (2) Age ≥18 years; (3) Normal results on comprehensive physical examination and absence of major chronic diseases; (4) Willingness to participate and provision of written informed consent.

Exclusion criteria: (1) Severe dysfunction of vital organs, including heart, liver, or kidneys; (2) Presence of malignant neoplasms or other life-threatening comorbidities; (3) Pregnancy or lactation; (4) Use of medications known to influence metabolic parameters within the past three months; (5) Other non-diabetic foot conditions (e.g., infectious or traumatic foot disorders); (6) Inability or unwillingness to comply with study procedures.

#### 2.1.2 Clinical data collection

All clinical data were meticulously extracted from the electronic medical record system in a standardized manner. The collected information was categorized as follows: Demographic and baseline characteristics: age, sex, heart rate, blood pressure, place of residence, occupation, smoking history, and alcohol consumption history. Diabetes-related parameters: duration of diabetes, anti-hyperglycemic treatment regimen before admission, and family history of diabetes. Foot ulcer characteristics (specific to the DF group): time from ulcer onset to clinical presentation, etiology, anatomical location, wound surface area, IWGDF/IDSA classification, and results of microbial culture of wound exudates. Comorbidities and complications: presence or absence of concomitant conditions, such as hypertension, cerebrovascular disease, cardiovascular disease, fatty liver, and chronic kidney disease.

Laboratory measurements: Fasting venous blood samples were collected for analysis, which included the following indicators:

Glucose metabolism: glycated hemoglobin (HbA1c) and fasting blood glucose (FBG).

Lipid profile: total cholesterol (TC), triglycerides (TG), high-density lipoprotein cholesterol (HDL-C), and low-density lipoprotein cholesterol (LDL-C).

Inflammatory markers: high-sensitivity C-reactive protein (hs-CRP), erythrocyte sedimentation rate (ESR), complete blood count including red blood cell (RBC) and white blood cell (WBC) counts with differential classification.

Coagulation profile: platelet count (PLT), fibrinogen (FIB), and D-dimer (D-Dimer).

Liver and renal function: albumin (ALB), total bilirubin (TBIL), direct bilirubin (DBIL), alanine aminotransferase (ALT), aspartate aminotransferase (AST), alkaline phosphatase (ALP), creatinine (Cr), blood urea nitrogen (BUN), and uric acid (UA).

Other laboratory tests: serum electrolytes (Na^+^, Ca^2+^;, K^+^), urine microalbumin, and urine albumin-to-creatinine ratio (UACR).

Imaging examinations: findings from cardiac ultrasound and vascular ultrasound of the neck and lower extremities.

### 2.2 Serum sample collection and non-targeted metabolomics analysis

#### 2.2.1 Sample pretreatment

All subjects fasted overnight and provided 5 mL of venous blood in the early morning. Blood samples were allowed to stand at room temperature for 30 minutes to facilitate coagulation, followed by centrifugation at 3,000 rpm for 10 minutes to separate serum. The resulting serum was aliquoted into cryotubes and immediately stored at -80°C until further analysis. For metabolomic profiling, 50 μL of serum was mixed with 200 μL of methanol-acetonitrile (1:1, v/v, containing internal standards) and vortexed for 30 seconds. The mixture was subjected to ultrasonication in an ice-water bath for 10 minutes to ensure complete protein denaturation and metabolite extraction. After incubation at -40°C for 1 hour to promote protein precipitation, the samples were centrifuged at 12,000 rpm and 4°C for 15 minutes. The supernatant was collected and used for injection. Additionally, a pooled quality control (QC) sample was prepared by mixing equal volumes of all individual samples, which was processed alongside experimental samples throughout the analytical run.

#### 2.2.2 Liquid chromatography-mass spectrometry analysis

Analyses were carried out using an ultra-high-performance liquid chromatography coupled with quadrupole-Orbitrap mass spectrometry (UHPLC-Q-Orbitrap MS) system. Chromatographic conditions: A Vanquish UHPLC system (Thermo Fisher Scientific, USA), equipped with a Waters ACQUITY UPLC BEH Amide column (2.1 mm × 50 mm, 1.7 μm), was utilized. The mobile phase was composed of solvent A (water containing 25 mM ammonium acetate and 25 mM ammonia water) and solvent B (acetonitrile). Gradient elution was implemented at a flow rate of 0.4 mL/min. The column temperature was kept at 40°C, and the injection volume was set to 2 μL.

Mass spectrometry conditions: An Orbitrap Exploris 120 mass spectrometer (Thermo Fisher Scientific) was operated in both positive and negative electrospray ionization (ESI) modes. Key source parameters were as follows: spray voltage, 3.8 kV (positive mode) or -3.4 kV (negative mode); sheath gas flow rate, 50 arbs; auxiliary gas flow rate, 15 arbs; capillary temperature, 320°C. Full-scan data were acquired in high-resolution mode with a mass range of m/z 50–1,000.

To ensure the reliability and stability of metabolomics data, this study implemented a rigorous quality control protocol. A pooled quality control (QC) sample was prepared by equally mixing all 54 serum samples. Within the UHPLC-MS/MS analytical sequence, one QC sample was analyzed after every 10 experimental samples, resulting in a total of 7 QC injections to monitor system performance throughout the run. Additionally, blank samples containing only the extraction solvent were intermittently analyzed to evaluate potential contamination. The baseline of the total ion chromatogram from the QC samples remained stable, indicating consistent chromatographic separation (**Supplementary Figures S1A, B**). The relative standard deviation (RSD) of retention times for the six internal standards was below 0.05%, and the median RSD of their peak areas was 2.36%, both within acceptable instrument stability criteria (**Supplementary Table S1**). For the final set of detected metabolites, the median RSD of peak areas in QC samples was 12.7%, which meets the established reliability threshold for untargeted metabolomics studies (RSD < 20%). Principal component analysis revealed tight clustering of all QC samples in the score plot, with pairwise correlation coefficients of metabolite expression exceeding 0.95, confirming high analytical reproducibility (**Supplementary Figures S1C, D**). Furthermore, no significant contaminants were observed in the blank sample analyses (**Supplementary Figures S1E, F**).

#### 2.2.3 Data processing and metabolite identification

Raw data files were transformed into mzXML format using ProteoWizard software (version 3.0). Subsequently, peak detection, alignment, retention time correction, and quantification were conducted using customized R scripts. To minimize technical variability and rectify potential batch effects resulting from instrument drift, data normalization was carried out based on the pooled QC samples analyzed intermittently throughout the sequence. Metabolites were identified by comparing accurate mass measurements (molecular ions) from the primary mass spectra and fragment ion patterns from tandem MS/MS spectra with the BiotreeDB database (version 3.0). A mass accuracy tolerance of ≤10 ppm was applied for molecular ion matching.

#### 2.2.4 Metabolomics data analysis

Normalized metabolite abundance data were subjected to multivariate statistical analysis. Unsupervised principal component analysis (PCA) was conducted using SIMCA-P software (version 14.1) to assess overall clustering trends and data distribution. This was followed by supervised orthogonal partial least squares-discriminant analysis (OPLS-DA) to maximize intergroup separation and identify discriminating variables. Model validity was confirmed via 200 permutation tests to evaluate overfitting risk. Differential metabolites were selected based on the following criteria: variable importance in projection (VIP) > 1.0, false discovery rate-adjusted p-value (q-value) < 0.05 from univariate t-tests, and fold change (FC) > 1.5 or < 0.67. Pathway enrichment analysis of significantly altered metabolites was performed using the MetaboAnalyst 5.0 web-based platform, based on the KEGG pathway database.

#### 2.2.5 Screening of protective factors

To identify potential protective metabolites against DF, binary logistic regression analyses were conducted based on the differentially expressed metabolites identified through metabolomics. First, multicollinearity among candidate metabolites was assessed using variance inflation factor (VIF) analysis; metabolites with VIF ≥ 5 were excluded. Next, univariate logistic regression (*P* < 0.05) was performed to screen significant predictors. Variables showing statistical significance in univariate analysis were then entered into a multivariate logistic regression model using the forward likelihood ratio (LR) method. Odds ratios (ORs) and corresponding 95% confidence intervals (CIs) were calculated. Metabolites associated with OR < 1 and *P* < 0.05 were considered statistically significant protective factors.

### 2.3 Cell function verification experiment

#### 2.3.1 Cell culture and model establishment

Human dermal fibroblasts (HSF) were purchased from Nanjing COBIOER Biotechnology Co., Ltd. (CBP60672). The cells were cultured in DMEM medium (normal glucose concentration of 5.5 mM) containing 10% fetal bovine serum and 1% penicillin-streptomycin in a 37°C, 5% CO_2_ incubator. To simulate the high glucose environment of diabetes, the cells were inoculated into high glucose DMEM medium (30 mM glucose) and cultured for 24 hours to establish the high glucose injury model. All other reagents were of domestic analytical purity.

#### 2.3.2 Experimental grouping and intervention

The experiment was divided into four groups:

1. Control group (NG): 5.5 mM glucose.

2. High glucose model group (HG): 30 mM glucose.

3. High sugar + CDCA intervention group: in a 30 mM glucose culture medium, 5, 10, and 25 nM CDCA (Shanghai yuanye Biology, B20347) were added respectively. CDCA was dissolved in dimethyl sulfoxide to ensure that the final concentration of DMSO in each experimental group was less than 0.1%.

#### 2.3.3 Cell proliferation assay

The CCK - 8 assay kit (CTCC, M006) was employed. HSF cells were plated at a density of 5×104 cells per well in a 96 - well plate. Following the intervention in accordance with the aforementioned groups for 24 hours, 10 μL of CCK - 8 solution was added to each well and then incubated for an additional 2 hours. The absorbance value at a wavelength of 450 nm was determined using an enzyme detector.

#### 2.3.4 Evaluation of cell migration ability

##### 2.3.4.1 Transwell

After the HSF cells were treated according to the above groups for 24 hours, 200 μL of cell suspension (5 × 10^4^ cells) was added to the upper chamber of the Transwell, and the lower chamber was filled with culture medium containing 10% FBS. After incubation for 24 hours, the cells that did not migrate in the upper chamber were wiped off with a cotton swab, and the cells in the lower chamber were fixed with 4% paraformaldehyde and stained with crystal violet. Five random fields were selected for counting under a microscope.

##### 2.3.4.2 Scratch test

The HSF cells were cultured in a 6-well plate until they reached 90% confluence. A scratch was created using a 200 μL sterile pipette. The plate was rinsed with PBS and then the culture medium of the corresponding group was added. Images were captured under a microscope at 0 hours and 24 hours. Subsequently, the change in scratch area was measured, and the healing rate was calculated.

#### 2.3.5 RNA extraction and real-time fluorescence quantitative PCR

The HSF cells receiving corresponding group intervention will be extracted for total RNA using the TRIzol method, and then reverse transcribed into cDNA. With GAPDH as the internal reference gene, amplification will be performed using SYBR Green (Beijing Aidel, PC3302) pre-mixed reagent on the Real-time PCR instrument (ABI, 7500). The relative mRNA expression levels of proliferating cell nuclear antigen (PCNA), α-smooth muscle actin (α-SMA), and vimentin will be calculated using the 2^(-ΔΔCt)^ method. The primer sequences used are as follows (**Table 1**).

**Table 1:** Primer sequences for real-time PCR.

Primers	Sequence (5’-3’)	PCR products size
PCNA (F)	CAAGAAGGTGTTGGAGGCA	153bp
PCNA (R)	TCGCAGCGGTAGGTGTC	
α-SMA (F)	GGTGATGGTGGGAATG	186bp
α-SMA (R)	AGGGTGGGATGCTCTT	
Vimentin (F)	GAACGCAAAGTGGAATC	134bp
Vimentin (R)	AGGTCAGGCTTGGAAA	
GAPDH (F)	CGGATTTGGTCGTATTG	151bp
GAPDH (R)	GAAGATGGTGATGGGATT	

#### 2.3.6 Western blotting

HSF cells were treated with different concentrations (5, 10, and 25 nM) of CDCA for 24 hours and then collected. Total protein was extracted using RIPA lysis buffer supplemented with protease and phosphatase inhibitors. The protein concentration was determined using a BCA protein assay kit.

Proteins were separated by 10% SDS-PAGE using equal amounts (30 μg) per sample and then transferred onto PVDF membranes. The membranes were blocked with 5% bovine serum albumin (BSA) for 2 h at room temperature, followed by overnight incubation at 4°C with primary antibodies specific to the target proteins. The primary antibodies used included FXR (Proteintech, 25055-1-AP, dilution 1:1000), p-PKA (Affinity, AF7246, 1:1000), AMPK (Affinity, AF6423, 1:1000), p-AMPK (Affinity, AF3423, 1:1000), and β-actin (Proteintech, 66009-1-Ig, 1:5000). Following several washes with TBST, the membranes were incubated with HRP-conjugated secondary antibodies (1:5000) for 1 h at room temperature under mild shaking. Protein signals were detected using an ECL chemiluminescence substrate and captured using the ChemiScope 5300 Pro imaging system. Densitometric analysis of the resulting bands was performed using ImageJ software.

### 2.4 Statistical analysis

Statistical analysis was conducted using SPSS 26.0 and GraphPad Prism 8.0 software. Regarding clinical data, continuous variables that follow a normal distribution are presented as the mean ± standard deviation, and comparisons among multiple groups were carried out using one-way analysis of variance (ANOVA). Data with non-normal distribution are expressed as the median (interquartile range), and comparisons were made using the Kruskal-Wallis H test. Categorical variables are reported as frequency (percentage) and were analyzed using the chi - square test or Fisher’s exact test, as appropriate. Cell-based experiments were independently repeated at least three times, and the data are presented as the mean ± standard deviation. One-way ANOVA followed by Tukey’s *post hoc* test for pairwise comparisons was employed for multi-group analyses. A *p*-value < 0.05 was regarded as statistically significant for all tests.

## 3 Results

### 3.1 Patients with DF exhibit significant metabolic disturbances, systemic inflammation, and vascular complications

To precisely characterize the clinical phenotypes of individuals with DF, we carried out a comprehensive and systematic comparison of baseline data across three groups: healthy controls (HA), patients with DM, and patients with DF. As presented in **Table 2**, no statistically significant differences were detected in fundamental demographic characteristics such as age and sex among the three groups (*P* > 0.05), which implies sufficient comparability between the groups. Nevertheless, the average length of hospital stay in the DF group was notably longer than that in the DM group (*P* < 0.01), and the percentage of rural residents was the highest in the DF group (56%, *P* = 0.046). This finding suggests that socioeconomic factors might exert a crucial influence on the clinical management of diabetic foot (DF) (**Table 2**).

**Table 2:** Baseline demographic and clinical characteristics.

Variable	HA (n = 18)	DM (n = 18)	DF (n = 18)	P-value
Demographics				
Age (years)	59.72 ± 2.08	58.5 ± 11.85	61.22 ± 11.07	0.481
Gender (Female), n (%)	9 (50.00)	9 (50.00)	10 (55.56)	0.929
Hospitalization				
Length of stay (days), median (IQR)	N/A	11 (7.5, 12)	19.5 (11.5, 31.5)	**0.002**
Residence & Socioeconomics				
Residence, n (%)	N/A			**0.046**
Urban	N/A	7 (38.89)	5 (27.78)	
County/Town	N/A	4 (22.22)	0 (0.00)	
Rural	N/A	7 (38.89)	13 (72.22)	
Lifestyle & Medical History				
Smoking, n (%)	N/A	5 (27.78)	6 (33.33)	1.000
Drinking, n (%)	N/A	6 (33.33)	5 (27.78)	1.000
Family History of Diabetes, n (%)	N/A	6 (33.33)	6 (33.33)	1.000
Diabetes-Related Characteristics				
Duration of Diabetes (months), median (IQR)	N/A	72 (7.5, 165)	138 (51, 207)	0.177
Glucose-Lowering Regimen, n (%)	N/A			0.264
Oral Medications	N/A	10 (55.56)	12 (66.67)	
Insulin	N/A	1 (5.56)	4 (22.22)	
Combination Therapy	N/A	4 (22.22)	1 (5.56)	
Irregular or No Treatment	N/A	3 (16.67)		

For continuous variables, values are presented as mean ± standard deviation if normally distributed; otherwise, median (interquartile range) is reported. Categorical variables are expressed as frequency (percentage). P values were calculated using one-way analysis of variance or the Kruskal-Wallis H test for continuous data, and chi-square test or Fisher’s exact test for categorical data, as appropriate. N/A denotes not applicable. The bold values indicate that the P value is less than 0.05, and the difference is statistically significant.

Regarding metabolic and inflammatory markers, the DF group showed characteristic dysregulation patterns (**Table 3**; **Supplementary Table S2**). Although the fasting blood glucose level in the DF group (8.54 ± 2.57 mmol/L) was lower than that in the DM group (9.86 ± 2.44 mmol/L), both values were significantly higher than those in the HA group (*P* < 0.001). The DF group also presented obvious lipid abnormalities, manifested as increased levels of triglycerides (TG), total cholesterol (TC), and low-density lipoprotein cholesterol (LDL-C), along with decreased high-density lipoprotein cholesterol (HDL-C) levels (*P* < 0.01). Notably, systemic inflammatory markers were significantly elevated in the DF group. The median level of high-sensitivity C-reactive protein (hs-CRP) reached 9.56 mg/L, which was significantly higher than that of the DM group (1.78 mg/L) and the HA group (*P* < 0.001). Meanwhile, the erythrocyte sedimentation rate (ESR), white blood cell count (WBC), and neutrophil count all increased significantly (*P* < 0.05). Coagulation profile analysis indicated that the levels of fibrinogen (FIB) and D-dimer in the DF group were markedly elevated compared with those in the other two groups (*P* < 0.001), suggesting a pronounced hypercoagulable state.

**Table 3:** Comparison of laboratory parameters among the three groups.

Parameter	HA (n = 18)	DM (n =18)	DF (n = 18)	P-value
Glucose Metabolism				
FPG (mmol/L)	4.73 ± 0.47	9.86 ± 2.44	8.54 ± 2.57	**< 0.001**
HbA1c (%)	N/A	9.84 ± 2.54	10.40 ± 2.80	0.535
Lipid Profile				
TG (mmol/L)	1.60 (1.20, 1.81)	2.36 (1.73, 3.00)	1.39 (1.25, 1.73)	**0.007**
TC (mmol/L)	5.56 ± 0.89	4.97 ± 1.23	4.10 ± 0.73	**< 0.001**
LDL-C(mmol/L)	3.07 ± 0.61	2.71 ± 0.95	2.25 ± 0.59	**0.006**
HDL-C (mmol/L)	1.20 (1.02, 1.32)	0.93 (0.82, 1.03)	0.92 (0.82, 1.12)	**0.003**
Systemic Inflammatory Markers				
hsCRP (mg/L)	N/A	1.78 (0.54, 6.00)	9.56 (6.16, 10.00)	**< 0.001**
WBC (×10^9^/L)	N/A	6.10 (4.16,6.79)	7.52 (6.68, 9.28)	**0.002**
N (×10^9^/L)	N/A	3.34 (2.39, 4.21)	5.60 (4.60, 6.65)	**< 0.001**
ESR (mm/h)	N/A	4 (3, 8.75)	68.5 (37.5, 120.0)	**< 0.001**
Coagulation Function				
FIB (g/L)	N/A	2.50 (2.42, 2.86)	5.30 (4.53, 6.23)	**< 0.001**
D-dimer (mg/L)	N/A	0.25 (0.19, 0.31)	0.44 (0.33, 0.73)	**0.002**

The bold values indicate that the P value is less than 0.05, and the difference is statistically significant.

With regard to complications, the DF group exhibited a markedly higher prevalence of vascular involvement (**Table 4**). Specifically, 94.44% of patients in the DF group presented with vascular lesions, a significantly greater proportion compared to 27.78% in the DM group (*P* < 0.001), with lower extremity vascular lesions being the most prevalent (77.78% *vs*. 16.67%, *P* < 0.001). Furthermore, the DF group demonstrated significantly higher rates of concomitant heart disease (77.78% *vs*. 22.22%, *P* = 0.002) and diabetic nephropathy (44.44% *vs*. 0%, *P* = 0.003) relative to the DM group.

**Table 4:** Comparison of complications and comorbidities between the DM and DF groups.

Complications and comorbidities	DM (n = 18)	DF (n = 18)	P-value
Diabetic Microvascular Complications			
Diabetic nephropathy, n (%)	0 (0.00)	8 (44.44)	**0.003**
Diabetic retinopathy, n (%)	2 (11.11)	8 (44.44)	0.060
Diabetic neuropathy, n (%)	15 (83.33)	17 (94.44)	0.603
Diabetic Macrovascular & Peripheral Vascular Complications			
Diabetic vascular disease (overall), n (%)	5 (27.78)	17 (94.44)	**< 0.001**
Lower extremity vascular disease, n (%)	3 (16.67)	14 (77.78)	**< 0.001**
Cervical vascular disease, n (%)	10 (55.56)	11 (61.11)	0.162†
Heart disease, n (%)	4 (22.22)	14 (77.78)	**0.002**
Cerebrovascular disease, n (%)	3 (16.67)	4 (22.22)	1.000
Other Comorbidities			
Hypertension, n (%)	3 (16.67)	8 (44.44)	0.146
Fatty liver, n (%)	10 (55.56)	3 (16.67)	**0.035** ^†^

^†^This variable includes a “not done” category, which was excluded from the statistical analysis prior to P value calculation. The bold values indicate that the P value is less than 0.05, and the difference is statistically significant.

### 3.2 Non-targeted metabolomics reveals characteristic metabolic profiles of DF and identifies key downregulated metabolites

**Figure 1:**
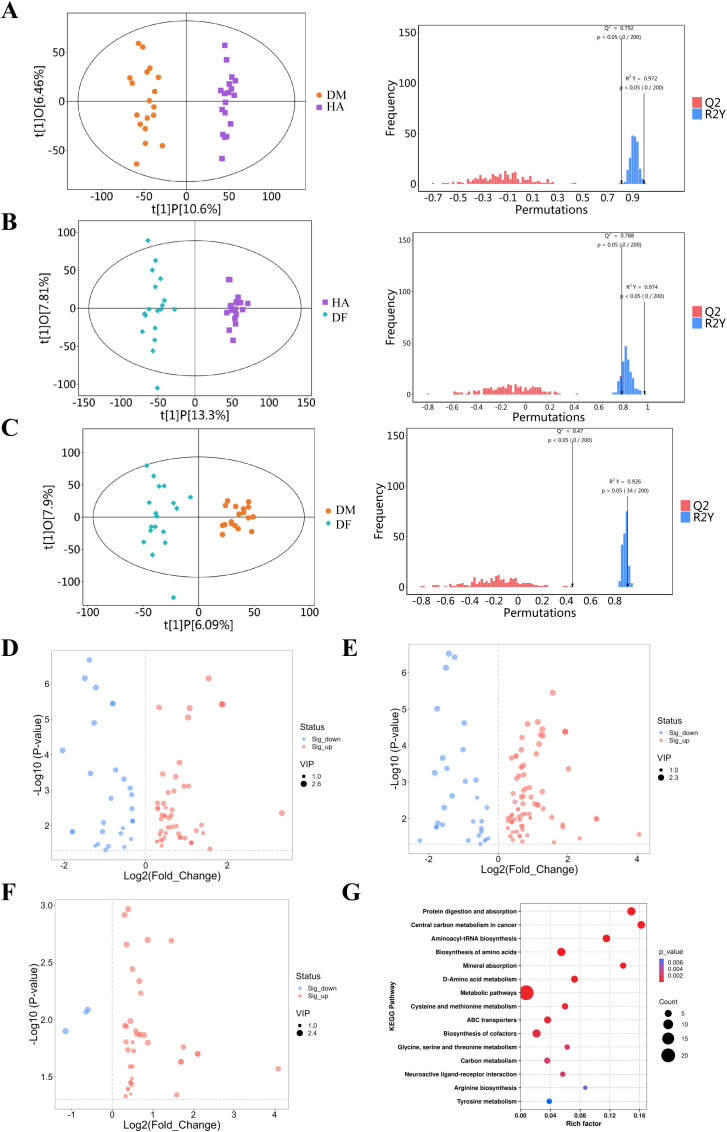
Untargeted metabolomic analysis of serum metabolic profiles among the three groups. **(A–C)** OPLS-DA score plots and permutation test plots: **(A)** HA *vs*. DM; **(B)** HA *vs*. DF; **(C)** DM *vs*. DF. The score plots reveal distinct metabolic separation trends among the three groups. The permutation test results (right, n=200 permutations) show that the intercepts of the R2Y and Q2 regression lines for all models are greater than 0.5, indicating the models are valid and not overfitted. **(D–F)** Volcano plot of differential metabolites: Each dot represents a metabolite. Gray dots represent metabolites with no significant difference; red dots represent metabolites significantly up-regulated in the DF group (VIP > 1.0, *P* < 0.05, FC > 1.5); blue dots represent metabolites significantly down-regulated in the DF group (VIP > 1.0, *P* < 0.05, FC < 0.67). Dashed lines indicate the thresholds for statistical significance and fold change. **(G)** Bubble chart of KEGG pathway enrichment analysis for differential metabolites between DM and DF groups: Bubble size represents the pathway impact value; bubble color represents the significance level of the enrichment (-log10(*P*-value)). The most significantly enriched pathways are labeled.

The quality of the non-targeted metabolomics data in this study remained stable and fully met the analytical requirements, as clearly evidenced by the tight clustering of quality control samples. Through the analysis of 54 serum samples from three groups, a metabolic dysregulation profile associated with DF was successfully constructed. The OPLS-DA models effectively demonstrated significant metabolic separation among the HA, DM, and DF groups (**Figures 1A–C**), and the validity of the models was confirmed by permutation testing (Q² > 0.5).

The results clearly demonstrated that a total of 80 differentially expressed metabolites were identified when comparing the HA and DM groups (51 up-regulated and 29 down-regulated; **Figure 1D**; **Supplementary Table S3**). Similarly, 106 differentially expressed metabolites were found between the HA and DF groups (76 up-regulated and 30 down-regulated; **Figure 1E**; **Supplementary Table S4**), and 41 were identified between the DM and DF groups (38 up-regulated and 3 down-regulated; **Figure 1F**; **Supplementary Table S5**). When closely examining the metabolic progression from DM to DF, specific attention was focused on the down-regulated metabolites. Three crucial down-regulated molecules were precisely identified: chenodeoxycholic acid (CDCA, FC = 0.66, VIP = 2.09, *P* = 0.008), 1-methyl nicotinamide (1 - MNA, FC = 0.64, VIP = 1.73, *P* = 0.009), and trimethylamine N-oxide (TMAO, FC = 0.45, VIP = 2.33, *P* = 0.013). Pathway enrichment analysis (**Figure 1G**) additionally revealed that the metabolic disturbances between the DM and DF groups were mainly associated with the ABC transporter pathway (impact factor: 21.74%), along with glycine, serine, and threonine metabolism; cysteine and methionine metabolism; and starch and sucrose metabolism, among other significant pathways.

### 3.3 Functional metabolomics and regression analysis jointly identify CDCA as a key protective factor for DF

To further assess the association between the aforementioned down-regulated metabolites and the risk of DF, a logistic regression analysis was conducted. Variance inflation factor (VIF) diagnostics demonstrated that the VIF values for CDCA, 1-MNA, and TMAO were all less than 5, suggesting no substantial multicollinearity among the predictor variables.

As presented in **Table 5**, univariate logistic regression analysis indicated that CDCA (OR = 0.918, 95% CI: 0.280–0.955, *P* = 0.035) and 1 - MNA (OR = 0.013, 95% CI: 0.000–0.451, *P* = 0.017) were significantly and inversely correlated with the risk of DF. In contrast, the association of TMAO with DF risk did not achieve statistical significance (*P* = 0.108). In the multivariate logistic regression model, both CDCA (OR = 0.429, 95% CI: 0.225–0.815, *P* = 0.010) and 1-MNA (OR = 0.033, 95% CI: 0.002–0.455, *P* = 0.011) remained independently and significantly associated with a reduced risk of DF, as evidenced by odds ratios less than 1 and 95% confidence intervals that did not include 1. Considering that CDCA plays well-established and multifaceted biological roles in regulating glucose and lipid metabolism, insulin sensitivity, and possesses antibacterial and anti-inflammatory properties, while the evidence for the role of 1-MNA is relatively scarce, CDCA was chosen as the primary candidate molecule for subsequent functional validation.

**Table 5:** Logistic regression analysis of differential metabolites for DF risk.

Metabolite	Univariate analysis	P-value	Multivariate analysis	P-value
	OR (95% CI)		OR (95% CI)	
CDCA	0.918 (0.280 - 0.955)	0.035	0.429 (0.225 - 0.815)	**0.010**
1-MNA	0.013 (0.000 - 0.451)	0.017	0.033 (0.002 - 0.455)	**0.011**
TMAO	0.697 (0.449 - 1.082)	0.108	–	

The bold values indicate that the P value is less than 0.05, and the difference is statistically significant.

### 3.4 Cell experiments confirm that CDCA reverses high glucose-induced fibroblast dysfunction

To validate the biological function of CDCA, HSFs were cultured under high glucose conditions (30 mM), and the effects of varying concentrations of CDCA on cellular function were assessed.

#### 3.4.1 CDCA enhances the proliferative capacity of HSFs under high glucose conditions

**Figure 2:**
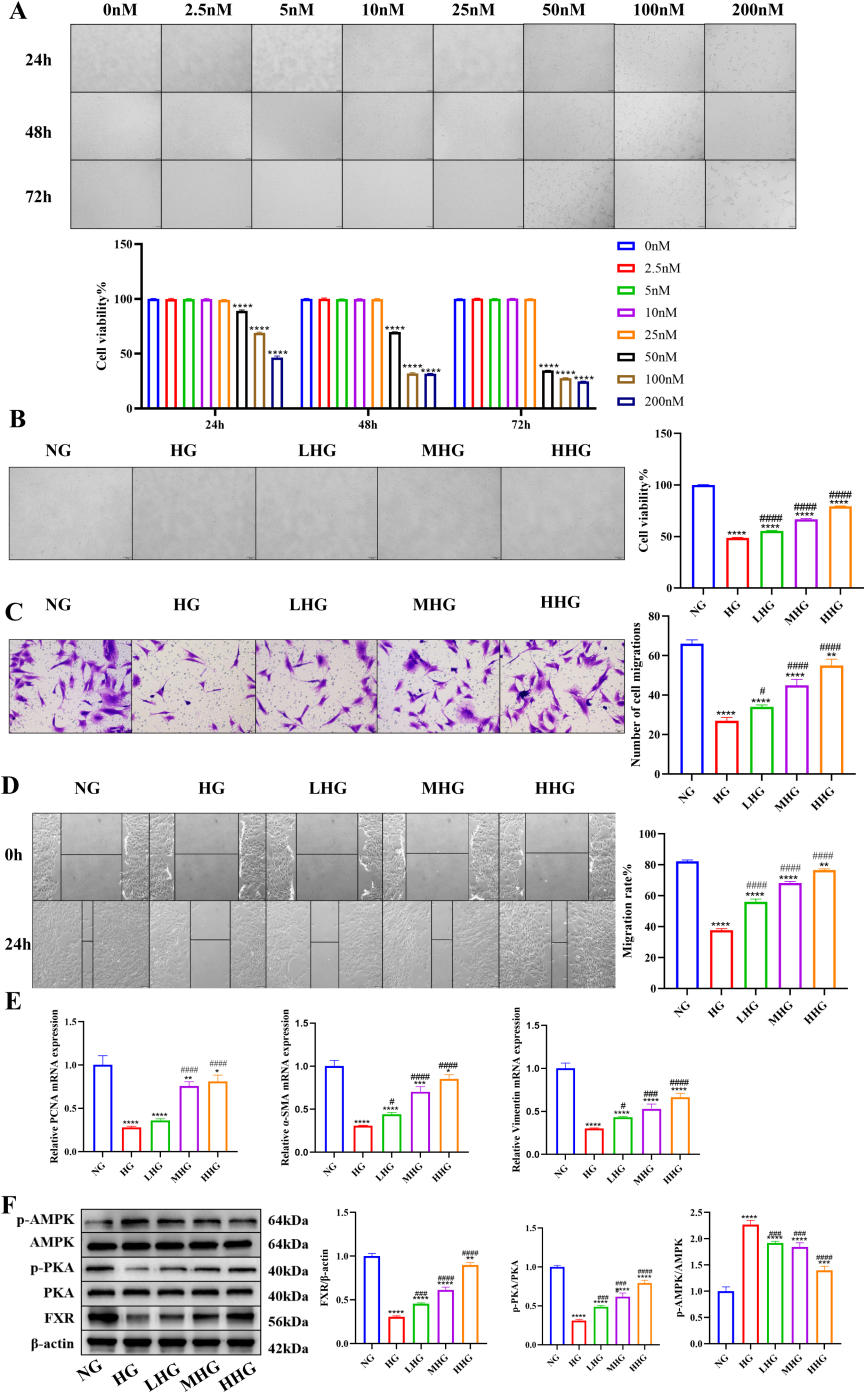
Effects of different concentrations of CDCA treatment on HSF cells. **(A)** Morphological observations and quantitative bar graph of HSF cells following treatment with various concentrations of CDCA over different time periods. ^*^Compared with the control group, ^*^P < 0.05, ^**^P < 0.01, ^***^P < 0.001, ^****^P < 0.0001. **(B)** Effect of CDCA concentration on the viability of HSF cells. **(C)** Effect of different CDCA concentrations on the number of migrating HSF cells. **(D)** Effect of different CDCA concentrations on the wound healing rate of HSF cells. **(E)** Relative mRNA expression levels of PCNA, α-SMA, Vimentin, and GAPDH in HSF cells after treatment with varying concentrations of CDCA, with GAPDH used as an internal reference. **(F)** Effects of CDCA on signaling pathway protein expression in HSF cells under high glucose conditions. ^*^Compared with the NG group, ^*^P < 0.05, ^**^P < 0.01, ^***^P < 0.001, ^****^P < 0.0001; ^#^Compared with the HG group, ^#^*P* < 0.05, ^##^*P* < 0.01, ^###^*P* < 0.001, ^####^*P* < 0.0001.

The effects of varying concentrations and treatment durations of CDCA on the proliferative capacity of HSFs were assessed using the CCK-8 assay. Results across time points were consistent: compared with the control group, treatment with 2.5 nM to 25 nM CDCA did not significantly affect cell viability, whereas exposure to 50 nM–200 nM CDCA led to a significant, dose-dependent reduction in viability. Therefore, 5 nM, 10 nM, and 25 nM CDCA were selected as low, medium, and high concentrations, respectively, for subsequent experiments, with a standardized treatment duration of 24 hours (**Figure 2A**; **Supplementary Figure S2**).

HSF cells were exposed to high glucose conditions with or without CDCA treatment. As shown in **Figure 2B**, cell viability in the high glucose model group was significantly reduced compared to the normal control group (*P* < 0.001). Notably, CDCA intervention attenuated this inhibitory effect in a dose-dependent manner.

#### 3.4.2 CDCA enhances the migration capacity of HSFs under high glucose conditions

The findings from the Transwell migration assay (**Figure 2C**) and the scratch wound healing assay (**Figure 2D**) consistently indicated that high-glucose treatment notably compromised the migratory ability of human skin fibroblasts (HSFs). When compared with the high-glucose (HG) group, the quantity of cells migrating across the Transwell membrane was significantly elevated in the CDCA intervention groups (*P* < 0.01). In the scratch wound healing assay, the wound closure rate was substantially enhanced after CDCA treatment, especially at a concentration of 25 nM, where it almost returned to normal levels (*P* < 0.001).

#### 3.4.3 CDCA upregulates mRNA expression of genes associated with tissue repair

The expression levels of key genes associated with cell proliferation and migration were evaluated by qRT-PCR (**Figure 2E**). The results indicated that under high glucose conditions, the mRNA expression of the proliferation marker PCNA, the myofibroblast differentiation marker α - SMA, and the cytoskeletal protein Vimentin was notably suppressed. After CDCA intervention, the mRNA expression of these genes was upregulated in a dose-dependent manner (PCNA: *P* < 0.001; α-SMA: *P* < 0.01; Vimentin: *P* < 0.05), which provided a molecular basis for the CDCA - mediated enhancement of cell proliferation and migration.

#### 3.4.4 CDCA exerts protective effects by activating the FXR/PKA/AMPK signaling pathway

To further elucidate the molecular mechanism through which CDCA reverses high glucose-induced fibroblast dysfunction, we examined the expression of key proteins in the FXR, PKA, and AMPK pathways, which are closely related to bile acid signaling and cellular energy metabolism (**Figure 2F**). Western blotting results indicated that, when compared with the normal control group, the high glucose model group showed significantly reduced FXR expression (*P* < 0.01), decreased p-PKA levels (*P* < 0.05), and a notable increase in p-AMPK levels (*P* < 0.001), suggesting suppressed bile acid signaling and enhanced cellular energy stress under hyperglycemic conditions. CDCA intervention upregulated FXR expression in a dose-dependent manner (*P* < 0.001). Simultaneously, p-PKA levels were significantly increased (*P* < 0.01), while p-AMPK activation was markedly inhibited (*P* < 0.001). These findings imply that CDCA may alleviate high glucose-induced fibroblast dysfunction by activating FXR and PKA signaling and inhibiting excessive AMPK activation.

## 4 Discussion

DFU represent one of the most severe complications associated with diabetes, presenting a substantial global public health burden. Epidemiological evidence indicates that the lifetime risk of foot ulcer development among individuals with diabetes may reach up to 34%, with an estimated annual global incidence of approximately 18.6 million cases (2, 27). More seriously, the mortality risk among patients with DFU is 2.5 times higher than that among diabetic patients without foot ulcers, and it is a leading cause of non-traumatic lower limb amputations in many countries (2). Approximately half of all DFUs develop secondary infections, which can potentially lead to sepsis, gangrene, and ultimately amputation. These findings jointly demonstrate the disease burden of DFU, which is marked by high incidence, high disability rate, and high mortality, along with the substantial complexity of its clinical management.

Currently, the clinical management primarily relies on a multidisciplinary collaborative approach, with its core components including vascular reconstruction, infection control, local ulcer management, and offloading therapy (28, 29). Although these strategies are crucial for limb preservation, they mainly concentrate on alleviating late-stage pathological consequences. The underlying pathophysiology of DF entails progressive tissue damage in the foot triggered by chronic hyperglycemia, which is characterized by a “neuro-vascular-immune” triad of dysfunction (30). Persistent hyperglycemia gives rise to peripheral neuropathy, which leads to the loss of protective sensation. It also facilitates the development of peripheral vascular disease, causing ischemia in the lower limbs. Coupled with immune dysfunction in a hyperglycemic milieu, these factors work in synergy to create a local microenvironment that hampers tissue repair and increases the susceptibility to infection. Therefore, transcending the traditional models of “anti-infective therapy” and “vascular revascularization” and concentrating on the analysis and targeted intervention of this pathological microenvironment has emerged as a promising avenue for enhancing outcomes in DF.

Against this backdrop, this study systematically clarifies the metabolic dysregulation characteristics of DFU by integrating clinical metabolomics and functional validation. Moreover, it identifies CDCA as a key endogenous protective factor in DF for the first time. The core finding reveals that CDCA is a disease-specific protective metabolite for DF, and its significantly decreased serum concentration is independently correlated with an increased risk of DF development. This discovery not only deepens our understanding of the metabolic basis of DF but also provides a promising approach for the development of novel therapeutic strategies based on metabolic modulation.

At the mechanistic level, this study verifies that CDCA effectively alleviates high glucose-induced cellular damage by activating the pathways related to cell proliferation and migration. *In vitro* experiments show that, within its physiological concentration range (5–25 nM), CDCA reverses the functional impairment in HSFs under high glucose conditions in a dose-dependent manner. This protective effect is accompanied by a significant up-regulation of the proliferation marker PCNA, the myofibroblast differentiation marker α-SMA, and the cytoskeletal protein vimentin (31, 32). These molecular alterations collectively form the mechanistic basis for CDCA-mediated enhancement of wound repair. This indicates that CDCA may expedite wound healing by facilitating entry into the proliferative phase, inducing differentiation into functional myofibroblasts, and maintaining cytoskeletal integrity. Moreover, this study demonstrated the regulatory impact of CDCA on the FXR/PKA/AMPK signaling pathway in cell models related to DF. The current results clearly showed that CDCA significantly reversed the high-glucose-induced downregulation of FXR expression and the inhibition of PKA phosphorylation. Simultaneously, it also mitigated the excessive activation of AMPK. FXR, as a central nuclear receptor for bile acids, promotes cellular proliferation and metabolic homeostasis upon activation. PKA is involved in the regulation of cell migration and gene transcription, and appropriate suppression of AMPK could contribute to alleviating the high-glucose-induced energy stress and apoptosis. These findings suggest that CDCA may enhance fibroblast proliferation, migration, and the expression of repair-related genes, and thus accelerate wound healing, by coordinately activating FXR and PKA while curbing the excessive activation of AMPK. Future studies employing gene silencing or receptor antagonists are needed to further verify the necessity of these pathways in mediating the effects of CDCA.

Although CDCA shows promising wound-repair potential *in vitro*, its stability, local permeability, and targeted delivery in complex wound environments require improvement. Nanocarrier-based drug delivery systems have advanced bioavailability, controlled release, and targeting capabilities. For instance, cubosomes—bicontinuous cubic liquid crystalline nanoparticles—enhance skin penetration and local retention of anti-inflammatory drugs and can be administered orally, transdermally, or intranasally, showing therapeutic potential in inflammatory conditions like rheumatoid arthritis (33). Similarly, transfersomes, as ultra-deformable lipid vesicles, effectively cross the stratum corneum and improve drug delivery to skin tissues, with proven efficacy for anti-inflammatory, antifungal, and peptide therapies (34). Therefore, encapsulating CDCA in nanocarriers such as cubosomes or transfersomes could enhance its stability, skin penetration, and sustained release at wound sites. This approach aligns with the strategy of “metabolic microenvironment remodeling” and may accelerate the translational development of CDCA-based treatments for DFU.

It should be noted that CDCA, as a crucial component of bile acids, exhibits multiple biological functions, such as the regulation of glucose and lipid metabolism homeostasis, anti - inflammatory effects, and antibacterial activity (31). This pleiotropic nature precisely meets the dual therapeutic requirements of infection control and metabolic improvement in the clinical management of DF. Previous studies have indicated that CDCA regulates glucose homeostasis and inflammatory responses by activating the FXR and TGR5 (21, 32). The current findings expand these mechanisms to the context of wound repair, providing a more comprehensive understanding of the protective role of CDCA in DF.

The innovative value of this study resides in the initial identification of CDCA as a potential therapeutic target for diabetic foot (DF) and the introduction of a novel treatment paradigm—metabolic microenvironment remodeling (35). Unlike conventional therapeutic strategies that predominantly concentrate on anti - infective therapy and vascular reconstruction, our findings indicate that enhancing the local metabolic environment at the wound site by supplementing endogenous protective metabolites could be a promising approach to surmounting the current treatment limitations in DF. This strategy directly tackles the fundamental metabolic dysfunction that is characteristic of DF and has substantial potential for clinical translation.

However, this study presents several limitations. Firstly, the single-center design and the restricted sample size might undermine the generalizability of the study’s findings. Future validation of the clinical value of CDCA necessitates multicenter, large-scale cohort studies (36). Secondly, the study mainly concentrated on cellular - level validation and was devoid of supporting evidence from animal models, thus restricting a comprehensive evaluation of CDCA’s therapeutic effects *in vivo*. Thirdly, the initial logistic regression model that identified CDCA as a protective factor failed to adjust for potential clinical confounders, such as the duration of diabetes or comorbid conditions. Although the comparability of the baseline groups alleviates this concern, future analyses using adjusted models are required to confirm the independent protective role of CDCA. More importantly, the specific molecular mechanisms underlying CDCA’s protective effects—particularly whether they are mediated through canonical bile acid receptors such as FXR and TGR5—remain to be thoroughly elucidated through experimental approaches including gene knockout (37, 38). Based on this, future research will focus on completing the construction of a complete evidence chain from *in vitro* to *in vivo*: 1) Utilizing gene editing technology, clarify in cell models whether CDCA exerts its effects through specific receptor pathways such as FXR and TGR5; 2) Establish an animal model of diabetic foot ulcers, and through local or systemic administration, evaluate the direct effects of CDCA on wound healing speed, tissue remodeling, and inflammatory regulation *in vivo*; 3) If the results are positive, further explore its preclinical efficacy and safety to lay the foundation for potential translational applications. This study also represents the first to demonstrate an independent negative association between serum CDCA levels and the risk of diabetic foot, while elucidating its direct protective effects at the cellular level—thereby providing preliminary etiological evidence supporting its potential as a novel biomarker. However, the evaluation of key performance indicators for CDCA as a diagnostic or predictive biomarker remains beyond the scope of this study, including the determination of optimal cut-off values for disease differentiation, sensitivity, specificity, and predictive value.

In conclusion, this study establishes the protective function of CDCA in DF and paves a new research path for DF treatment by targeting the metabolic microenvironment. Future endeavors ought to concentrate on clarifying the specific mechanism of action of CDCA and validating its therapeutic effectiveness in more intricate in - vivo models, with the ultimate aim of promoting the clinical translation of metabolic regulation - based therapeutic strategies for DF.

## 5 Conclusion

This study systematically characterizes the metabolic dysregulation features of DFU via the integration of clinical metabolomics and functional validation, and for the first time, it identifies CDCA as a crucial endogenous protective factor. The findings indicate that the serum CDCA levels in patients with DF are significantly lower compared to those in both uncomplicated diabetic patients and healthy controls. Moreover, multivariate logistic regression analysis verifies that CDCA is an independent protective factor against the progression of DF. Further cellular functional experiments reveal that CDCA effectively reverses high-glucose-induced fibroblast dysfunction in a dose-dependent manner within the physiological concentration range (5–25 nM), and the underlying mechanisms are closely related to enhanced cell proliferation, improved migratory capacity, and upregulation of repair-related genes such as PCNA and α-SMA. In conclusion, this study not only clarifies the protective role and underlying molecular mechanisms of CDCA in the pathogenesis of DF but also offers a solid experimental basis and potential therapeutic targets for the development of innovative strategies targeting the metabolic microenvironment.

## Data Availability

The original contributions presented in the study are included in the article/**Supplementary Material**. Further inquiries can be directed to the corresponding authors.
