# Supplementary material for: Metabolomics identifies chenodeoxycholic acid as a protective factor in diabetic foot ulcers
Source: Front Endocrinol (Lausanne). 2026 Feb 13;17:1754743. doi: 10.3389/fendo.2026.1754743 (PMC12945834; doi:10.3389/fendo.2026.1754743)
Supplement: Supplementary file 1 [file DataSheet1.docx]

**
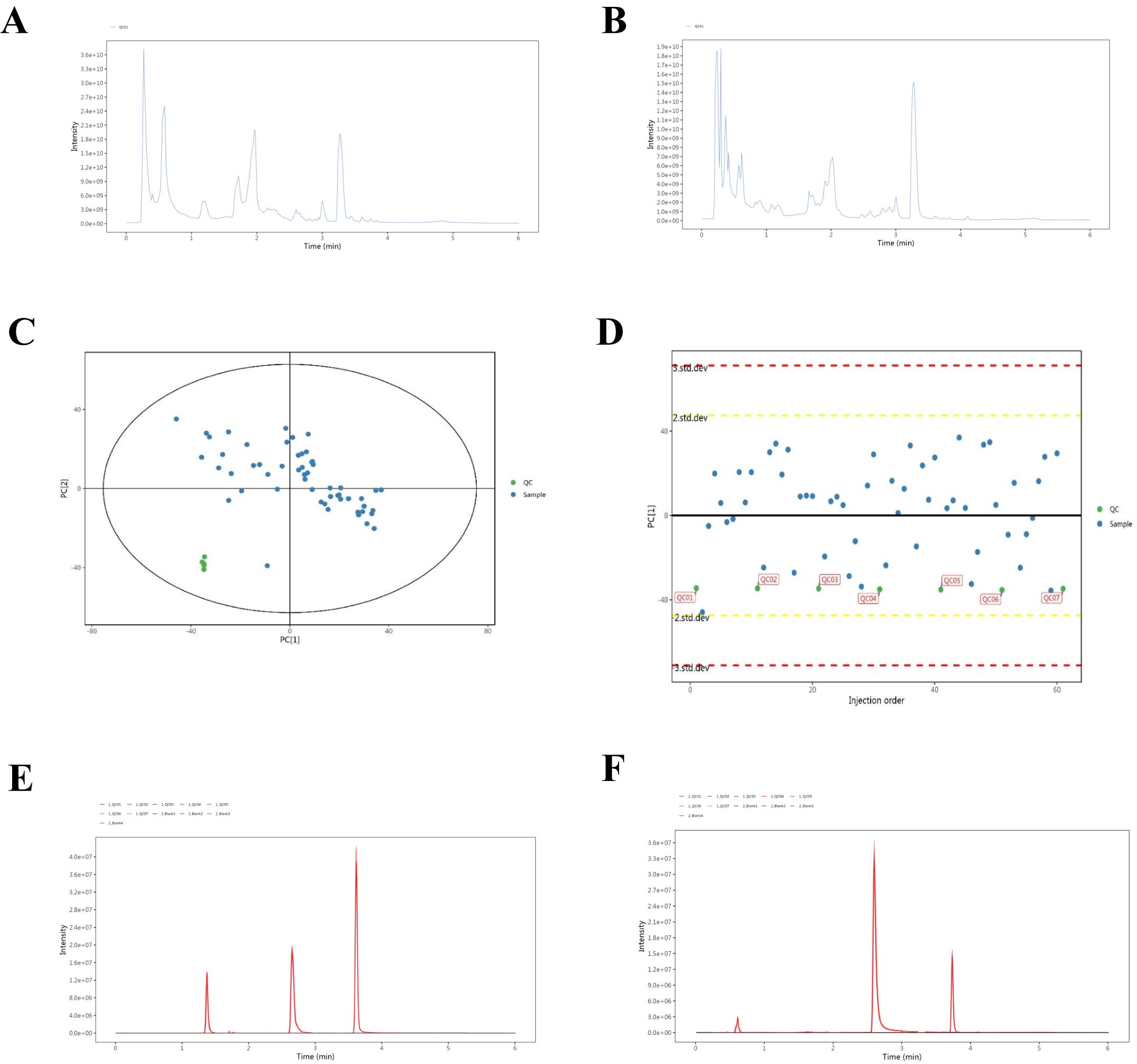
Supplementary Figure S1 Quality control related to metabolomics.**（A）TIC chart of QC sample detection in positive ion mode (B) TIC diagram of QC sample detection in negative ion mode.(C) PCA Score Plot.(D)1D Distribution Plot of PCA-X for QC Samples. (E)EIC of internal standards in blank samples and QC under positive ion mode .(F) EIC of internal standards in blank samples and QC under negative ion mode.

**Supplementary Figure S2 Morphological changes in HSF cells treated with CDCA at varying concentrations and time points.**

**Supplementary Table 1. Stability of Internal Standard Responses in Quality Control Samples**

| **Internal Standard ID** | **Retention time（RT）** | **Mass-to-charge ratio（m/z）** | **Relative standard deviation（RSD）** |
| --- | --- | --- | --- |
| IS1 | 36.5 | 142.0604 | 0.01032 |
| IS2 | 224.3 | 121.0444 | 0.0142 |
| IS3 | 156.3 | 133.1061 | 0.0236 |
| IS4 | 82.6 | 155.174 | 0.0179 |
| IS5 | 159.6 | 127.1427 | 0.0202 |
| IS6 | 217.5 | 110.1082 | 0.0534 |

**Supplementary Table 2**

**Comparison of Laboratory Parameters Among the Three Groups**

| Parameter | HA (n = 18) | DM (n = 18) | DF (n = 18) | P-value |
| --- | --- | --- | --- | --- |
| **Vital Signs** |  |  |  |  |
| Heart Rate (bpm) | N/A | 84.22 ± 10.88 | 87.17 ± 15.52 | 0.515 |
| Systolic BP (mmHg) | 138.56 ± 15.82 | 128.11 ± 11.98 | 131.28 ± 22.89 | 0.195 |
| Diastolic BP (mmHg) | 82.06 ± 9.03 | 79.89 ± 7.10 | 79.44 ± 13.02 | 0.707 |
| **Glucose Metabolism** |  |  |  |  |
| FPG (mmol/L) | 4.73 ± 0.47 | 9.86 ± 2.44 | 8.54 ± 2.57 | **< 0.001** |
| HbA1c (%) | N/A | 9.84 ± 2.54 | 10.40 ± 2.80 | 0.535 |
| **Lipid Profile** |  |  |  |  |
| TG (mmol/L) | 1.60 (1.20, 1.81) | 2.36 (1.73, 3.00) | 1.39 (1.25, 1.73) | **0.007** |
| TC (mmol/L) | 5.56 ± 0.89 | 4.97 ± 1.23 | 4.10 ± 0.73 | **< 0.001** |
| LDL-C (mmol/L) | 3.07 ± 0.61 | 2.71 ± 0.95 | 2.25 ± 0.59 | **0.006** |
| HDL-C (mmol/L) | 1.20 (1.02, 1.32) | 0.93 (0.82, 1.03) | 0.92 (0.82, 1.12) | **0.003** |
| **Systemic Inflammatory Markers** |  |  |  |  |
| hsCRP (mg/L) | N/A | 1.78 (0.54, 6.00) | 9.56 (6.16, 10.00) | **< 0.001** |
| WBC (×10⁹/L) | N/A | 6.10 (4.16,6.79) | 7.52 (6.68, 9.28) | **0.002** |
| N (×10⁹/L) | N/A | 3.34 (2.39, 4.21) | 5.60 (4.60, 6.65) | **< 0.001** |
| L (×10⁹/L) | N/A | 1.77 ± 0.56 | 1.51 ± 0.50 | 0.149 |
| M (×10⁹/L) | N/A | 0.41 ± 0.18 | 0.48 ± 0.17 | 0.231 |
| NLR | N/A | 1.87 (1.41, 2.57) | 3.49 (2.56, 5.35) | **< 0.001** |
| ESR (mm/h) | N/A | 4 (3, 8.75) | 68.5 (37.5, 120.0) | **< 0.001** |
| **Hematology** |  |  |  |  |
| RBC (×10¹²/L) | N/A | 4.51 ± 0.62 | 3.95 ± 0.77 | **0.023** |
| Hb (g/L) | N/A | 136.89 ± 16.78 | 116.56 ± 22.20 | **0.004** |
| PLT (×10⁹/L) | N/A | 195 (167.5, 218.0) | 268.5 (241.75, 368.5) | **< 0.001** |
| **Coagulation Function** |  |  |  |  |
| FIB (g/L) | N/A | 2.50 (2.42, 2.86) | 5.30 (4.53, 6.23) | **< 0.001** |
| D-dimer (mg/L) | N/A | 0.25 (0.19, 0.31) | 0.44 (0.33, 0.73) | **0.002** |
| **Renal Function** |  |  |  |  |
| Cr (μmol/L) | N/A | 59.5 (53.5, 67.45) | 55.05 (45.75, 70.25) | 0.776 |
| UREA (mmol/L) | 5.05 (4.45, 5.70) | 5.85 (5.20, 6.80) | 5.80 (5.05, 7.80) | **0.046** |
| UA (μmol/L) | 327.22 ± 61.77 | 291.87 ± 87.69 | 277.17 ± 75.16 | 0.135 |
| Urine microalbumin (mg/L) | N/A | 5.39 (4.05, 9.93) | 42.60 (12.36, 201.72) | **0.001** |
| ACR (mg/g) | N/A | 5.88 (4.50, 8.51) | 23.36 (4.35, 131.55) | **0.048** |
| **Liver Function** |  |  |  |  |
| ALB (g/L) | N/A | 41.36 ± 3.81 | 38.27 ± 4.67 | **0.037** |
| ALT (U/L) | N/A | 17.5 (15.0, 26.0) | 15.5 (12.0, 22.0) | 0.211 |
| AST (U/L) | N/A | 17 (14.0, 21.5) | 18 (13.0, 22.25) | 0.874 |
| ALP (U/L) | N/A | 74 (61.5, 90.25) | 69 (55.25, 85.0) | 0.342 |
| TBIL (μmol/L) | N/A | 7.60 (6.62, 10.00) | 7.50 (5.58, 14.97) | 0.740 |
| DBIL (μmol/L) | N/A | 2.94 ± 1.00 | 3.35 ± 1.34 | 0.305 |
| **Electrolytes** |  |  |  |  |
| Na⁺ (mmol/L) | N/A | 138.51 ± 2.32 | 138.44 ± 4.50 | 0.952 |
| K⁺ (mmol/L) | N/A | 3.83 ± 0.36 | 4.19 ± 0.56 | **0.028** |
| Ca²⁺ (mmol/L) | N/A | 2.29 ± 0.14 | 2.30 ± 0.16 | 0.897 |
| **Cardiac Function** |  |  |  |  |
| LVEF (%) | N/A | 61.5 (59.25, 62.0) |  | 0.001 |

**Supplementary Table 3.**

**Selected Significant Differential Serum Metabolites Between HA and DM Groups**

| Category | Metabolite Name | VIP | P-value | Fold Change | Trend (DM vs HA) |
| --- | --- | --- | --- | --- | --- |
| **Bile Acids** |  |  |  |  |  |
|  | Glycocholic acid | 1.17 | 0.016 | 2.65 | ↑ |
|  | Taurochenodeoxycholic acid | 1.34 | 0.030 | 2.17 | ↑ |
| **Amino Acids & Metabolism** |  |  |  |  |  |
|  | N-Acetylhistamine | 2.17 | 0.004 | 10.10 | ↑ |
|  | Proline | 1.58 | 0.001 | 1.62 | ↑ |
|  | Ornithine | 1.62 | 0.003 | 0.79 | ↓ |
|  | Aspartate | 1.58 | 0.001 | 0.79 | ↓ |
| **Saccharides** |  |  |  |  |  |
|  | Mannose | 2.59 | < 0.001 | 3.67 | ↑ |
|  | Glucose | 2.59 | < 0.001 | 3.67 | ↑ |
|  | Fructose | 2.48 | < 0.001 | 2.92 | ↑ |
|  | 1,5-Anhydroglucitol | 2.21 | < 0.001 | 0.24 | ↓ |
| **Lipids & Fatty Acids** |  |  |  |  |  |
|  | 3-Hydroxybutyric acid | 1.50 | 0.014 | 2.36 | ↑ |
|  | Acetylcarnitine (Car(2:0)) | 1.62 | 0.001 | 2.07 | ↑ |
|  | Lauroylcarnitine (Car(12:0)) | 2.08 | < 0.001 | 0.42 | ↓ |
|  | Docosahexaenoic acid (DHA) | 1.65 | 0.008 | 0.49 | ↓ |
| **Energy Metabolism & Nucleosides** |  |  |  |  |  |
|  | Inosine | 1.84 | < 0.001 | 0.38 | ↓ |
|  | Guanosine | 2.25 | < 0.001 | 0.36 | ↓ |
|  | Nicotinamide | 1.74 | < 0.001 | 0.60 | ↓ |
|  | 1-Methylnicotinamide | 1.41 | 0.038 | 0.53 | ↓ |

Supplementary Table 4**.**

**Selected Significant Differential Serum Metabolites Between HA and DF Groups**

| Category | Metabolite Name | VIP | P-value | Fold Change | Trend (DF vs HA) |
| --- | --- | --- | --- | --- | --- |
| **Bile Acids** |  |  |  |  |  |
|  | **Chenodeoxycholic acid (CDCA)** | 1.64 | 0.001 | 0.64 | ↓ |
|  | 胆红素 Bilirubin | 1.35 | 0.040 | 0.55 | ↓ |
| **Amino Acids & Metabolism** |  |  |  |  |  |
|  | Proline | 2.11 | < 0.001 | 2.41 | ↑ |
|  | Arginine | 1.37 | 0.002 | 1.65 | ↑ |
|  | N-Acetylhistamine | 1.09 | 0.035 | 5.07 | ↑ |
|  | Ornithine | 1.04 | 0.035 | 0.82 | ↓ |
| **Saccharides** |  |  |  |  |  |
|  | Mannose | 2.14 | < 0.001 | 3.80 | ↑ |
|  | Glucose | 2.14 | < 0.001 | 3.80 | ↑ |
|  | Fructose | 2.15 | < 0.001 | 2.96 | ↑ |
|  | 1,5-Anhydroglucitol | 1.62 | < 0.001 | 0.36 | ↓ |
| **Lipids & Fatty Acids** |  |  |  |  |  |
|  | Acetylcarnitine (Car(2:0)) | 1.89 | < 0.001 | 2.33 | ↑ |
|  | Palmitoyl sphingomyelin | 1.61 | < 0.001 | 1.30 | ↑ |
|  | Lauroylcarnitine (Car(12:0)) | 2.11 | < 0.001 | 0.35 | ↓ |
|  | Docosahexaenoic acid (DHA) | 1.85 | 0.002 | 0.40 | ↓ |
| **Energy Metabolism, Nucleosides & Vitamins** |  |  |  |  |  |
|  | Inosine | 1.80 | < 0.001 | 0.42 | ↓ |
|  | Guanosine | 1.84 | < 0.001 | 0.51 | ↓ |
|  | Nicotinamide | 1.09 | 0.003 | 0.66 | ↓ |
|  | **1-Methylnicotinamide (1-MNA)** | 1.84 | 0.005 | 0.34 | ↓ |

Supplementary Table 5  **Serum Differential Metabolites Between DM and DF Groups**

| Category | Metabolite Name | VIP | P-value | Fold Change | Trend (DF vs DM) |
| --- | --- | --- | --- | --- | --- |
| **Amino Acids & Metabolism** |  |  |  |  |  |
|  | Proline | 1.74 | 0.012 | 1.49 | ↑ |
|  | Arginine | 1.36 | 0.016 | 1.41 | ↑ |
|  | Aspartate | 2.07 | 0.004 | 1.41 | ↑ |
|  | 甘氨酸 Glycine | 1.93 | 0.018 | 1.31 | ↑ |
|  | 丝氨酸 Serine | 1.71 | 0.011 | 1.27 | ↑ |
| **Gut Microbiota-Related Metabolites** |  |  |  |  |  |
|  | Phenyllactic acid | 2.06 | 0.023 | 3.24 | ↑ |
|  | Indolelactic acid | 1.83 | 0.006 | 1.63 | ↑ |
|  | p-Cresol sulfate | 2.06 | 0.020 | 4.31 | ↑ |
|  | **Trimethylamine N-oxide (TMAO)** | 2.33 | 0.013 | 0.45 | ↓ |
| **Bile Acids** |  |  |  |  |  |
|  | **Chenodeoxycholic acid (CDCA)** | 2.09 | 0.008 | 0.66 | ↓ |
| **Energy Metabolism & Nucleosides** |  |  |  |  |  |
|  | **1-Methylnicotinamide (1-MNA)** | 1.73 | 0.009 | 0.64 | ↓ |
